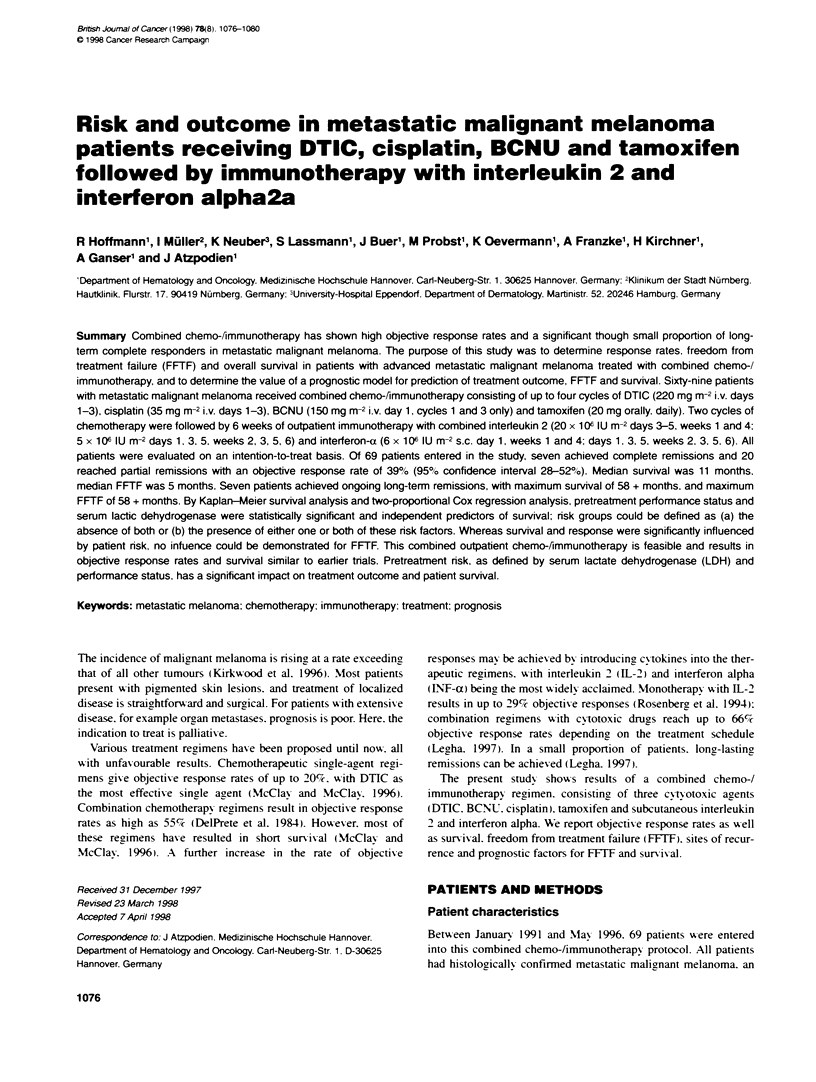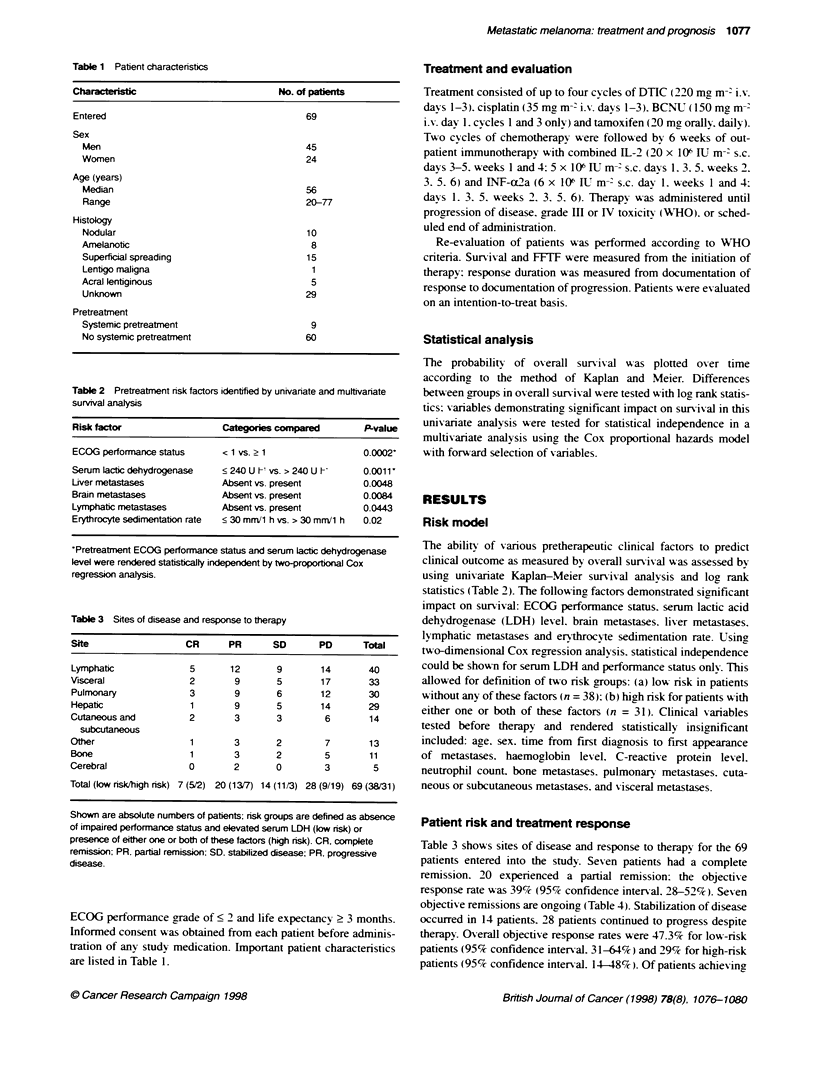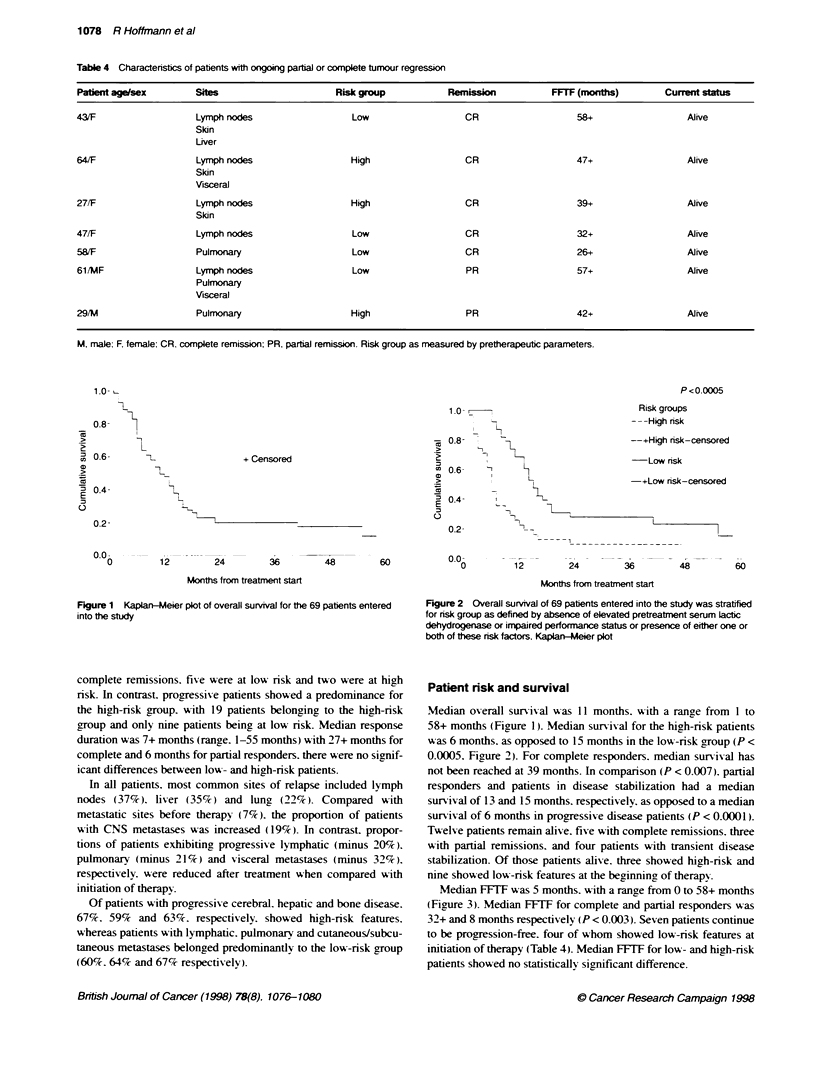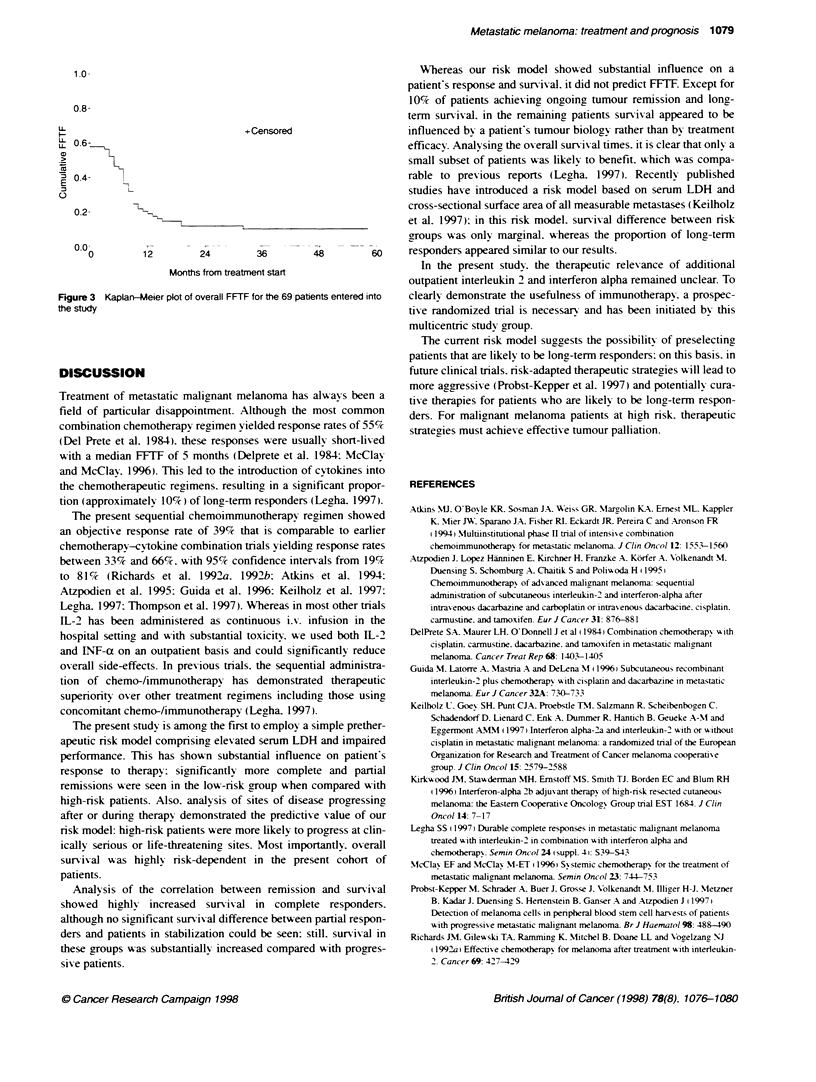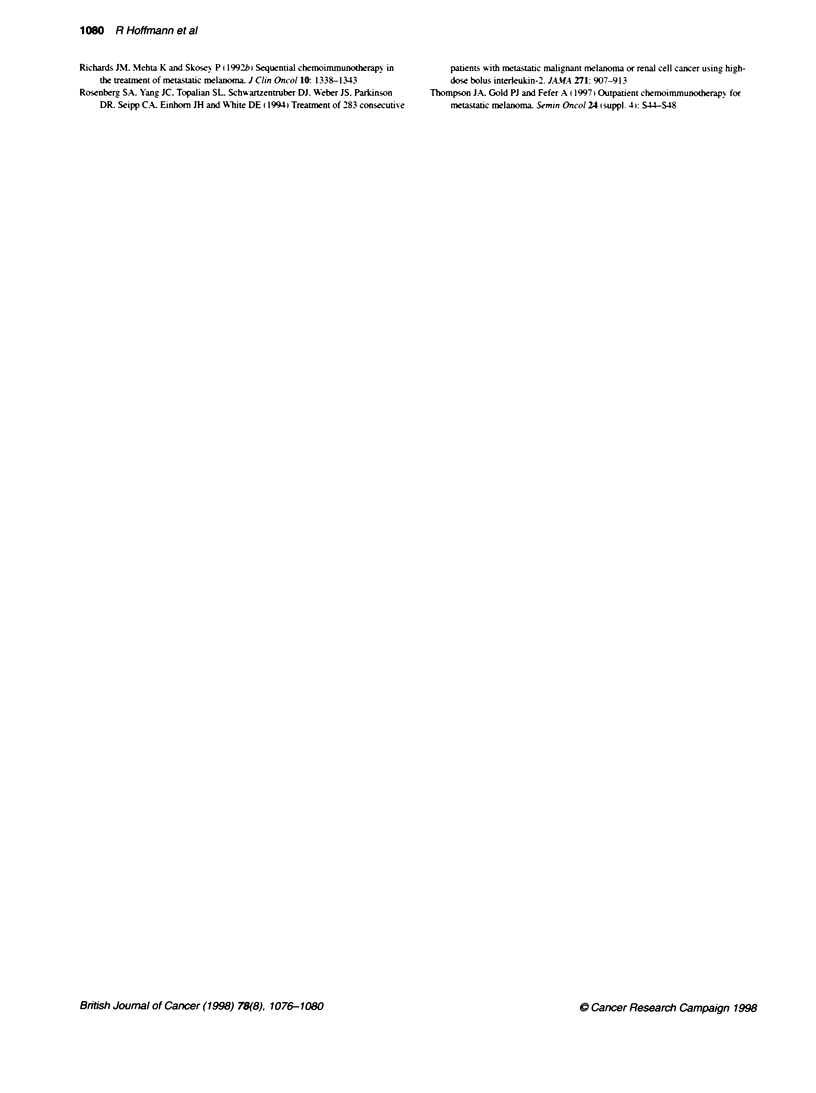# Risk and outcome in metastatic malignant melanoma patients receiving DTIC, cisplatin, BCNU and tamoxifen followed by immunotherapy with interleukin 2 and interferon alpha2a.

**DOI:** 10.1038/bjc.1998.630

**Published:** 1998-10

**Authors:** R. Hoffmann, I. Müller, K. Neuber, S. Lassmann, J. Buer, M. Probst, K. Oevermann, A. Franzke, H. Kirchner, A. Ganser, J. Atzpodien

**Affiliations:** Department of Hematology and Oncology, Medizinische Hochschule Hannover, Germany.

## Abstract

Combined chemo-/immunotherapy has shown high objective response rates and a significant though small proportion of long-term complete responders in metastatic malignant melanoma. The purpose of this study was to determine response rates, freedom from treatment failure (FFTF) and overall survival in patients with advanced metastatic malignant melanoma treated with combined chemo-/immunotherapy, and to determine the value of a prognostic model for prediction of treatment outcome, FFTF and survival. Sixty-nine patients with metastatic malignant melanoma received combined chemo-/immunotherapy consisting of up to four cycles of DTIC (220 mg m(-2) i.v. days 1-3), cisplatin (35 mg m(-2) i.v. days 1-3), BCNU (150 mg m(-2) i.v. day 1, cycles 1 and 3 only) and tamoxifen (20 mg orally, daily). Two cycles of chemotherapy were followed by 6 weeks of outpatient immunotherapy with combined interleukin 2 (20 x 10(6) IU m(-2) days 3-5, weeks 1 and 4; 5 x 10(6) IU m(-2) days 1, 3, 5, weeks 2, 3, 5, 6) and interferon-alpha (6 x 10(6) IU m(-2) s.c. day 1, weeks 1 and 4; days 1, 3, 5, weeks 2, 3, 5, 6). All patients were evaluated on an intention-to-treat basis. Of 69 patients entered in the study, seven achieved complete remissions and 20 reached partial remissions with an objective response rate of 39% (95% confidence interval 28-52%). Median survival was 11 months, median FFTF was 5 months. Seven patients achieved ongoing long-term remissions, with maximum survival of 58 + months, and maximum FFTF of 58 + months. By Kaplan-Meier survival analysis and two-proportional Cox regression analysis, pretreatment performance status and serum lactic dehydrogenase were statistically significant and independent predictors of survival; risk groups could be defined as (a) the absence of both or (b) the presence of either one or both of these risk factors. Whereas survival and response were significantly influenced by patient risk, no influence could be demonstrated for FFTF. This combined outpatient chemo-/immunotherapy is feasible and results in objective response rates and survival similar to earlier trials. Pretreatment risk, as defined by serum lactate dehydrogenase (LDH) and performance status, has a significant impact on treatment outcome and patient survival.


British Journal of Cancer (1998) 78(8), 1076–1080
© 1998 Cancer Research Campaign

### Risk and outcome in metastatic malignant melanoma patients receiving DTIC, cisplatin, BCNU and tamoxifen followed by immunotherapy with interleukin 2 and interferon alpha2a

**R Hoffmann[1], I Müller[2], K Neuber[3], S Lassmann[1], J Buer[1], M Probst[1], K Oevermann[1], A Franzke[1], H Kirchner[1], A Ganser[1] and J Atzpodien[1]**

[1]Department of Hematology and Oncology, Medizinische Hochschule Hannover, Carl-Neuberg-Str. 1, 30625 Hannover, Germany; [2]Klinikum der Stadt Nürnberg, Hautklinik, Flurstr. 17, 90419 Nürnberg, Germany; [3]University-Hospital Eppendorf, Department of Dermatology, Martinistr. 52, 20246 Hamburg, Germany

**Summary** Combined chemo-/immunotherapy has shown high objective response rates and a significant though small proportion of long-term complete responders in metastatic malignant melanoma. The purpose of this study was to determine response rates, freedom from treatment failure (FFTF) and overall survival in patients with advanced metastatic malignant melanoma treated with combined chemo-/immunotherapy, and to determine the value of a prognostic model for prediction of treatment outcome, FFTF and survival. Sixty-nine patients with metastatic malignant melanoma received combined chemo-/immunotherapy consisting of up to four cycles of DTIC (220 mg $m^{-2}$ i.v. days 1–3), cisplatin (35 mg $m^{-2}$ i.v. days 1–3), BCNU (150 mg $m^{-2}$ i.v. day 1, cycles 1 and 3 only) and tamoxifen (20 mg orally, daily). Two cycles of chemotherapy were followed by 6 weeks of outpatient immunotherapy with combined interleukin 2 ($20 \times 10^6$ IU $m^{-2}$ days 3–5, weeks 1 and 4; $5 \times 10^6$ IU $m^{-2}$ days 1, 3, 5, weeks 2, 3, 5, 6) and interferon-α ($6 \times 10^6$ IU $m^{-2}$ s.c. day 1, weeks 1 and 4; days 1, 3, 5, weeks 2, 3, 5, 6). All patients were evaluated on an intention-to-treat basis. Of 69 patients entered in the study, seven achieved complete remissions and 20 reached partial remissions with an objective response rate of 39% (95% confidence interval 28–52%). Median survival was 11 months, median FFTF was 5 months. Seven patients achieved ongoing long-term remissions, with maximum survival of 58 + months, and maximum FFTF of 58 + months. By Kaplan–Meier survival analysis and two-proportional Cox regression analysis, pretreatment performance status and serum lactic dehydrogenase were statistically significant and independent predictors of survival; risk groups could be defined as (a) the absence of both or (b) the presence of either one or both of these risk factors. Whereas survival and response were significantly influenced by patient risk, no infuence could be demonstrated for FFTF. This combined outpatient chemo-/immunotherapy is feasible and results in objective response rates and survival similar to earlier trials. Pretreatment risk, as defined by serum lactate dehydrogenase (LDH) and performance status, has a significant impact on treatment outcome and patient survival.

**Keywords:** metastatic melanoma; chemotherapy; immunotherapy; treatment; prognosis

The incidence of malignant melanoma is rising at a rate exceeding that of all other tumours (Kirkwood et al, 1996). Most patients present with pigmented skin lesions, and treatment of localized disease is straightforward and surgical. For patients with extensive disease, for example organ metastases, prognosis is poor. Here, the indication to treat is palliative.

Various treatment regimens have been proposed until now, all with unfavourable results. Chemotherapeutic single-agent regimens give objective response rates of up to 20%, with DTIC as the most effective single agent (McClay and McClay, 1996). Combination chemotherapy regimens result in objective response rates as high as 55% (DelPrete et al, 1984). However, most of these regimens have resulted in short survival (McClay and McClay, 1996). A further increase in the rate of objective responses may be achieved by introducing cytokines into the therapeutic regimens, with interleukin 2 (IL-2) and interferon alpha (INF-α) being the most widely acclaimed. Monotherapy with IL-2 results in up to 29% objective responses (Rosenberg et al, 1994); combination regimens with cytotoxic drugs reach up to 66% objective response rates depending on the treatment schedule (Legha, 1997). In a small proportion of patients, long-lasting remissions can be achieved (Legha, 1997).

The present study shows results of a combined chemo-/immunotherapy regimen, consisting of three cytyotoxic agents (DTIC, BCNU, cisplatin), tamoxifen and subcutaneous interleukin 2 and interferon alpha. We report objective response rates as well as survival, freedom from treatment failure (FFTF), sites of recurrence and prognostic factors for FFTF and survival.

*Received 31 December 1997*
*Revised 23 March 1998*
*Accepted 7 April 1998*

*Correspondence to:* J Atzpodien, Medizinische Hochschule Hannover, Department of Hematology and Oncology, Carl-Neuberg-Str. 1, D-30625 Hannover, Germany

### PATIENTS AND METHODS

### Patient characteristics

Between January 1991 and May 1996, 69 patients were entered into this combined chemo-/immunotherapy protocol. All patients had histologically confirmed metastatic malignant melanoma, an

**Table 1** Patient characteristics

| Characteristic | No. of patients |
|---|---|
| Entered | 69 |
| Sex | |
| Men | 45 |
| Women | 24 |
| Age (years) | |
| Median | 56 |
| Range | 20–77 |
| Histology | |
| Nodular | 10 |
| Amelanotic | 8 |
| Superficial spreading | 15 |
| Lentigo maligna | 1 |
| Acral lentiginous | 5 |
| Unknown | 29 |
| Pretreatment | |
| Systemic pretreatment | 9 |
| No systemic pretreatment | 60 |

**Table 2** Pretreatment risk factors identified by univariate and multivariate survival analysis

| Risk factor | Categories compared | P-value |
|---|---|---|
| ECOG performance status | < 1 vs. ≥ 1 | 0.0002* |
| Serum lactic dehydrogenase | ≤ 240 U $l^{-1}$ vs. > 240 U $l^{-1}$ | 0.0011* |
| Liver metastases | Absent vs. present | 0.0048 |
| Brain metastases | Absent vs. present | 0.0084 |
| Lymphatic metastases | Absent vs. present | 0.0443 |
| Erythrocyte sedimentation rate | ≤ 30 mm/1 h vs. > 30 mm/1 h | 0.02 |

*Pretreatment ECOG performance status and serum lactic dehydrogenase level were rendered statistically independent by two-proportional Cox regression analysis.

**Table 3** Sites of disease and response to therapy

| Site | CR | PR | SD | PD | Total |
|---|---|---|---|---|---|
| Lymphatic | 5 | 12 | 9 | 14 | 40 |
| Visceral | 2 | 9 | 5 | 17 | 33 |
| Pulmonary | 3 | 9 | 6 | 12 | 30 |
| Hepatic | 1 | 9 | 5 | 14 | 29 |
| Cutaneous and subcutaneous | 2 | 3 | 3 | 6 | 14 |
| Other | 1 | 3 | 2 | 7 | 13 |
| Bone | 1 | 3 | 2 | 5 | 11 |
| Cerebral | 0 | 2 | 0 | 3 | 5 |
| Total (low risk/high risk) | 7 (5/2) | 20 (13/7) | 14 (11/3) | 28 (9/19) | 69 (38/31) |

Shown are absolute numbers of patients; risk groups are defined as absence of impaired performance status and elevated serum LDH (low risk) or presence of either one or both of these factors (high risk). CR, complete remission; PR, partial remission; SD, stabilized disease; PR, progressive disease.

ECOG performance grade of ≤ 2 and life expectancy ≥ 3 months. Informed consent was obtained from each patient before administration of any study medication. Important patient characteristics are listed in Table 1.

### Treatment and evaluation

Treatment consisted of up to four cycles of DTIC (220 mg $m^{-2}$ i.v. days 1–3), cisplatin (35 mg $m^{-2}$ i.v. days 1–3), BCNU (150 mg $m^{-2}$ i.v. day 1, cycles 1 and 3 only) and tamoxifen (20 mg orally, daily). Two cycles of chemotherapy were followed by 6 weeks of outpatient immunotherapy with combined IL-2 (20 × $10^6$ IU $m^{-2}$ s.c. days 3–5, weeks 1 and 4; 5 × $10^6$ IU $m^{-2}$ s.c. days 1, 3, 5, weeks 2, 3, 5, 6) and INF-α2a (6 × $10^6$ IU $m^{-2}$ s.c. day 1, weeks 1 and 4; days 1, 3, 5, weeks 2, 3, 5, 6). Therapy was administered until progression of disease, grade III or IV toxicity (WHO), or scheduled end of administration.

Re-evaluation of patients was performed according to WHO criteria. Survival and FFTF were measured from the initiation of therapy; response duration was measured from documentation of response to documentation of progression. Patients were evaluated on an intention-to-treat basis.

### Statistical analysis

The probability of overall survival was plotted over time according to the method of Kaplan and Meier. Differences between groups in overall survival were tested with log rank statistics; variables demonstrating significant impact on survival in this univariate analysis were tested for statistical independence in a multivariate analysis using the Cox proportional hazards model with forward selection of variables.

### RESULTS

### Risk model

The ability of various pretherapeutic clinical factors to predict clinical outcome as measured by overall survival was assessed by using univariate Kaplan–Meier survival analysis and log rank statistics (Table 2). The following factors demonstrated significant impact on survival: ECOG performance status, serum lactic acid dehydrogenase (LDH) level, brain metastases, liver metastases, lymphatic metastases and erythrocyte sedimentation rate. Using two-dimensional Cox regression analysis, statistical independence could be shown for serum LDH and performance status only. This allowed for definition of two risk groups: (a) low risk in patients without any of these factors ($n$ = 38); (b) high risk for patients with either one or both of these factors ($n$ = 31). Clinical variables tested before therapy and rendered statistically insignificant included: age, sex, time from first diagnosis to first appearance of metastases, haemoglobin level, C-reactive protein level, neutrophil count, bone metastases, pulmonary metastases, cutaneous or subcutaneous metastases, and visceral metastases.

### Patient risk and treatment response

Table 3 shows sites of disease and response to therapy for the 69 patients entered into the study. Seven patients had a complete remission, 20 experienced a partial remission; the objective response rate was 39% (95% confidence interval, 28–52%). Seven objective remissions are ongoing (Table 4). Stabilization of disease occurred in 14 patients, 28 patients continued to progress despite therapy. Overall objective response rates were 47.3% for low-risk patients (95% confidence interval, 31–64%) and 29% for high-risk patients (95% confidence interval, 14–48%). Of patients achieving

© Cancer Research Campaign 1998

**Table 4** Characteristics of patients with ongoing partial or complete tumour regression

| Patient age/sex | Sites | Risk group | Remission | FFTF (months) | Current status |
|---|---|---|---|---|---|
| 43/F | Lymph nodes<br>Skin<br>Liver | Low | CR | 58+ | Alive |
| 64/F | Lymph nodes<br>Skin<br>Visceral | High | CR | 47+ | Alive |
| 27/F | Lymph nodes<br>Skin | High | CR | 39+ | Alive |
| 47/F | Lymph nodes | Low | CR | 32+ | Alive |
| 58/F | Pulmonary | Low | CR | 26+ | Alive |
| 61/MF | Lymph nodes<br>Pulmonary<br>Visceral | Low | PR | 57+ | Alive |
| 29/M | Pulmonary | High | PR | 42+ | Alive |

M, male; F, female; CR, complete remission; PR, partial remission. Risk group as measured by pretherapeutic parameters.

**Figure 1** Kaplan–Meier plot of overall survival for the 69 patients entered into the study

**Figure 2** Overall survival of 69 patients entered into the study was stratified for risk group as defined by absence of elevated pretreatment serum lactic dehydrogenase or impaired performance status or presence of either one or both of these risk factors. Kaplan–Meier plot complete remissions, five were at low risk and two were at high risk. In contrast, progressive patients showed a predominance for the high-risk group, with 19 patients belonging to the high-risk group and only nine patients being at low risk. Median response duration was 7+ months (range, 1–55 months) with 27+ months for complete and 6 months for partial responders, there were no significant differences between low- and high-risk patients.

In all patients, most common sites of relapse included lymph nodes (37%), liver (35%) and lung (22%). Compared with metastatic sites before therapy (7%), the proportion of patients with CNS metastases was increased (19%). In contrast, proportions of patients exhibiting progressive lymphatic (minus 20%), pulmonary (minus 21%) and visceral metastases (minus 32%), respectively, were reduced after treatment when compared with initiation of therapy.

Of patients with progressive cerebral, hepatic and bone disease, 67%, 59% and 63%, respectively, showed high-risk features, whereas patients with lymphatic, pulmonary and cutaneous/subcutaneous metastases belonged predominantly to the low-risk group (60%, 64% and 67% respectively).

### Patient risk and survival

Median overall survival was 11 months, with a range from 1 to 58+ months (Figure 1). Median survival for the high-risk patients was 6 months, as opposed to 15 months in the low-risk group ($P <$ 0.0005, Figure 2). For complete responders, median survival has not been reached at 39 months. In comparison ($P < 0.007$), partial responders and patients in disease stabilization had a median survival of 13 and 15 months, respectively, as opposed to a median survival of 6 months in progressive disease patients ($P < 0.0001$). Twelve patients remain alive, five with complete remissions, three with partial remissions, and four patients with transient disease stabilization. Of those patients alive, three showed high-risk and nine showed low-risk features at the beginning of therapy.

Median FFTF was 5 months, with a range from 0 to 58+ months (Figure 3). Median FFTF for complete and partial responders was 32+ and 8 months respectively ($P < 0.003$). Seven patients continue to be progression-free, four of whom showed low-risk features at initiation of therapy (Table 4). Median FFTF for low- and high-risk patients showed no statistically significant difference.

© Cancer Research Campaign 1998

**Figure 3** Kaplan–Meier plot of overall FFTF for the 69 patients entered into the study

### DISCUSSION

Treatment of metastatic malignant melanoma has always been a field of particular disappointment. Although the most common combination chemotherapy regimen yielded response rates of 55% (Del Prete et al. 1984), these responses were usually short-lived with a median FFTF of 5 months (Delprete et al. 1984; McClay and McClay, 1996). This led to the introduction of cytokines into the chemotherapeutic regimens, resulting in a significant proportion (approximately 10%) of long-term responders (Legha, 1997).

The present sequential chemoimmunotherapy regimen showed an objective response rate of 39% that is comparable to earlier chemotherapy–cytokine combination trials yielding response rates between 33% and 66%, with 95% confidence intervals from 19% to 81% (Richards et al. 1992*a*, 1992*b*; Atkins et al. 1994; Atzpodien et al. 1995; Guida et al. 1996; Keilholz et al. 1997; Legha, 1997; Thompson et al. 1997). Whereas in most other trials IL-2 has been administered as continuous i.v. infusion in the hospital setting and with substantial toxicity, we used both IL-2 and INF-α on an outpatient basis and could significantly reduce overall side-effects. In previous trials, the sequential administration of chemo-/immunotherapy has demonstrated therapeutic superiority over other treatment regimens including those using concomitant chemo-/immunotherapy (Legha, 1997).

The present study is among the first to employ a simple pretherapeutic risk model comprising elevated serum LDH and impaired performance. This has shown substantial influence on patient's response to therapy; significantly more complete and partial remissions were seen in the low-risk group when compared with high-risk patients. Also, analysis of sites of disease progressing after or during therapy demonstrated the predictive value of our risk model; high-risk patients were more likely to progress at clinically serious or life-threatening sites. Most importantly, overall survival was highly risk-dependent in the present cohort of patients.

Analysis of the correlation between remission and survival showed highly increased survival in complete responders, although no significant survival difference between partial responders and patients in stabilization could be seen; still, survival in these groups was substantially increased compared with progressive patients.

Whereas our risk model showed substantial influence on a patient's response and survival, it did not predict FFTF. Except for 10% of patients achieving ongoing tumour remission and long-term survival, in the remaining patients survival appeared to be influenced by a patient's tumour biology rather than by treatment efficacy. Analysing the overall survival times, it is clear that only a small subset of patients was likely to benefit, which was comparable to previous reports (Legha, 1997). Recently published studies have introduced a risk model based on serum LDH and cross-sectional surface area of all measurable metastases (Keilholz et al. 1997); in this risk model, survival difference between risk groups was only marginal, whereas the proportion of long-term responders appeared similar to our results.

In the present study, the therapeutic relevance of additional outpatient interleukin 2 and interferon alpha remained unclear. To clearly demonstrate the usefulness of immunotherapy, a prospective randomized trial is necessary and has been initiated by this multicentric study group.

The current risk model suggests the possibility of preselecting patients that are likely to be long-term responders; on this basis, in future clinical trials, risk-adapted therapeutic strategies will lead to more aggressive (Probst-Kepper et al. 1997) and potentially curative therapies for patients who are likely to be long-term responders. For malignant melanoma patients at high risk, therapeutic strategies must achieve effective tumour palliation.

### REFERENCES

Atkins MJ, O'Boyle KR, Sosman JA, Weiss GR, Margolin KA, Ernest ML, Kappler K, Mier JW, Sparano JA, Fisher RI, Eckardt JR, Pereira C and Aronson FR (1994) Multiinstitutional phase II trial of intensive combination chemoimmunotherapy for metastatic melanoma. *J Clin Oncol* **12**: 1553–1560

Atzpodien J, Lopez Hänninen E, Kirchner H, Franzke A, Körfer A, Volkenandt M, Duensing S, Schomburg A, Chaitik S and Poliwoda H (1995) Chemoimmunotherapy of advanced malignant melanoma: sequential administration of subcutaneous interleukin-2 and interferon-alpha after intravenous dacarbazine and carboplatin or intravenous dacarbacine, cisplatin, carmustine, and tamoxifen. *Eur J Cancer* **31**: 876–881

DelPrete SA, Maurer LH, O'Donnell J et al (1984) Combination chemotherapy with cisplatin, carmustine, dacarbazine, and tamoxifen in metastatic malignant melanoma. *Cancer Treat Rep* **68**: 1403–1405

Guida M, Latorre A, Mastria A and DeLena M (1996) Subcutaneous recombinant interleukin-2 plus chemotherapy with cisplatin and dacarbazine in metastatic melanoma. *Eur J Cancer* **32A**: 730–733

Keilholz U, Goey SH, Punt CJA, Proebstle TM, Salzmann R, Scheibenbogen C, Schadendorf D, Lienard C, Enk A, Dummer R, Hantich B, Geueke A-M and Eggermont AMM (1997) Interferon alpha-2a and interleukin-2 with or without cisplatin in metastatic malignant melanoma: a randomized trial of the European Organization for Research and Treatment of Cancer melanoma cooperative group. *J Clin Oncol* **15**: 2579–2588

Kirkwood JM, Stawderman MH, Ernstoff MS, Smith TJ, Borden EC and Blum RH (1996) Interferon-alpha 2b adjuvant therapy of high-risk resected cutaneous melanoma: the Eastern Cooperative Oncology Group trial EST 1684. *J Clin Oncol* **14**: 7–17

Legha SS (1997) Durable complete responses in metastatic malignant melanoma treated with interleukin-2 in combination with interferon alpha and chemotherapy. *Semin Oncol* **24** (suppl. 4): S39–S43

McClay EF and McClay M-ET (1996) Systemic chemotherapy for the treatment of metastatic malignant melanoma. *Semin Oncol* **23**: 744–753

Probst-Kepper M, Schrader A, Buer J, Grosse J, Volkenandt M, Illiger H-J, Metzner B, Kadar J, Duensing S, Hertenstein B, Ganser A and Atzpodien J (1997) Detection of melanoma cells in peripheral blood stem cell harvests of patients with progressive metastatic malignant melanoma. *Br J Haematol* **98**: 488–490

Richards JM, Gilewski TA, Ramming K, Mitchel B, Doane LL and Vogelzang NJ (1992*a*) Effective chemotherapy for melanoma after treatment with interleukin-2. *Cancer* **69**: 427–429

Richards JM, Mehta K and Skosey P (1992*b*) Sequential chemoimmunotherapy in the treatment of metastatic melanoma. *J Clin Oncol* **10**: 1338–1343

Rosenberg SA, Yang JC, Topalian SL, Schwartzentruber DJ, Weber JS, Parkinson DR, Seipp CA, Einhorn JH and White DE (1994) Treatment of 283 consecutive patients with metastatic malignant melanoma or renal cell cancer using high-dose bolus interleukin-2. *JAMA* **271**: 907–913

Thompson JA, Gold PJ and Fefer A (1997) Outpatient chemoimmunotherapy for metastatic melanoma. *Semin Oncol* **24** (suppl. 4): S44–S48

*© Cancer Research Campaign 1998*